# Novel loss-of-function mutation in *HERC2* is associated with severe developmental delay and paediatric lethality

**DOI:** 10.1136/jmedgenet-2020-106873

**Published:** 2020-06-22

**Authors:** Marilena Elpidorou, Sunayna Best, James A Poulter, Verity Hartill, Emma Hobson, Eamonn Sheridan, Colin A Johnson

**Affiliations:** 1 Division of Molecular Medicine, Leeds Institute of Medical Research, University of Leeds, Leeds, West Yorkshire, UK; 2 Yorkshire Clinical Genetics Service, Chapel Allerton Hospital, Leeds, West Yorkshire, UK

**Keywords:** developmental, molecular genetics

## Abstract

**Background:**

The *HERC2* gene encodes a 527 kDa E3 ubiquitin protein ligase that has key roles in cell cycle regulation, spindle formation during mitosis, mitochondrial functions and DNA damage responses. It has essential roles during embryonic development, particularly for neuronal and muscular functions. To date, missense mutations in *HERC2* have been associated with an autosomal recessive neurodevelopmental disorder with some phenotypical similarities to Angelman syndrome, and a homozygous deletion spanning *HERC2* and *OCA2* causing a more severe neurodevelopmental phenotype.

**Methods and results:**

We ascertained a consanguineous family with a presumed autosomal recessive severe neurodevelopmental disorder that leads to paediatric lethality. In affected individuals, we identified a homozygous *HERC2* frameshift variant that results in a premature stop codon and complete loss of HERC2 protein. Functional characterisation of this variant in fibroblasts, from one living affected individual, revealed impaired mitochondrial network and function as well as disrupted levels of known interacting proteins such as XPA.

**Conclusion:**

This study extends the genotype–phenotype correlation for *HERC2* variants to include a distinct lethal neurodevelopmental disorder, highlighting the importance of further characterisation for *HERC2*-related disorders.

## Introduction

Inherited neurodevelopmental disorders are a group of conditions with extensive genetic heterogeneity that present in the perinatal and paediatric age ranges and arise from a disruption in the development of the central nervous system. Common phenotypical characteristics shared by the majority of neurodevelopmental disorders include developmental delay, impaired motor function and intellectual disability. Structural brain abnormalities such as microcephaly and gyral defects are also observed in neurodevelopmental conditions. These phenotypes have considerable overlap and variability and can be indistinguishable on clinical grounds. A specific genetic diagnosis for an inherited neurodevelopmental disorder therefore enables accurate genetic counselling and better clinical management of patients and their families.

The HECT and RLD domain-containing E3 ubiquitin protein ligase 2 (*HERC2*) gene encodes a 527 kDa E3 ubiquitin protein ligase of multiple structural domains, including the cell cycle regulator RCC1 (RLD; Regulator of Chromosome Condensation 1-like domain) and C-terminal HECT (Homologous to the E6-AP Carboxyl Terminus) domains that are shared by various E3 ubiquitin protein ligases.^1^ The exact functions of HERC2 are still incompletely understood, but recent evidence suggests HERC2 targets key DNA damage response proteins, such as XPA and BRCA1, for degradation through the ubiquitin–proteasome system.^2 3^ XPA (Xeroderma pigmentosum complementation group A protein) is a DNA damage recognition protein that plays a key role in the nucleotide excision repair (NER) mechanism, and its correct turnover by HERC2 can significantly affect the circadian oscillation of NER activity, particularly in the brain.^4^ HERC2 also interacts with another E3 ubiquitin protein ligase, UBE3A, which when mutated has been shown to cause Angelman syndrome.^5^ Other important protein–protein interactions of HERC2 include proteins that are involved in cell cycle regulation, spindle formation during mitosis^6^ and mitochondrial bioenergetics.^7^

Two pathogenic missense variants in *HERC2* have been reported in individuals with developmental syndromes. Twenty-two individuals from three different Amish/Mennonite families homozygous for p.Pro594Leu were affected with autosomal recessive mental retardation type 38 (MIM 615516), described as an autism spectrum disorder phenotype with some similarities to Angelman syndrome.^8 9^ Common features included speech and language delay, gross motor delay, autistic features, seizures and childhood hypotonia. Two individuals have been reported with homozygous p.Arg1542His variants, with similar clinical features.^7^ Furthermore, a study has reported a single patient, presenting with severe developmental abnormalities and ocular albinism, who carries a homozygous deletion of the contiguous genes *HERC2* and *OCA2* resulting in a fusion protein.^10^

In this study, we describe a family presenting with a non-specific severe neurodevelopmental disorder characterised by profound developmental delay, blindness and perinatal mortality. We identified a homozygous frameshift variant in *HERC2* as the causative mutation in affected individuals, resulting in a lack of HERC2 protein. To our knowledge, this is the first report of a specific *HERC2* null allele causing a more severe neurodevelopmental disorder that does not resemble Angelman syndrome. This expands the genotype–phenotype correlations for *HERC2*-related disorders.

## Materials and methods

### Patients

Genomic DNA was obtained from blood samples using a standard salt precipitation protocol. Skin punch biopsies (4–6 mm) were obtained under local anaesthesia from a single affected child of the family (II:6).

### Whole-exome sequencing (WES)

**Figure 1:**
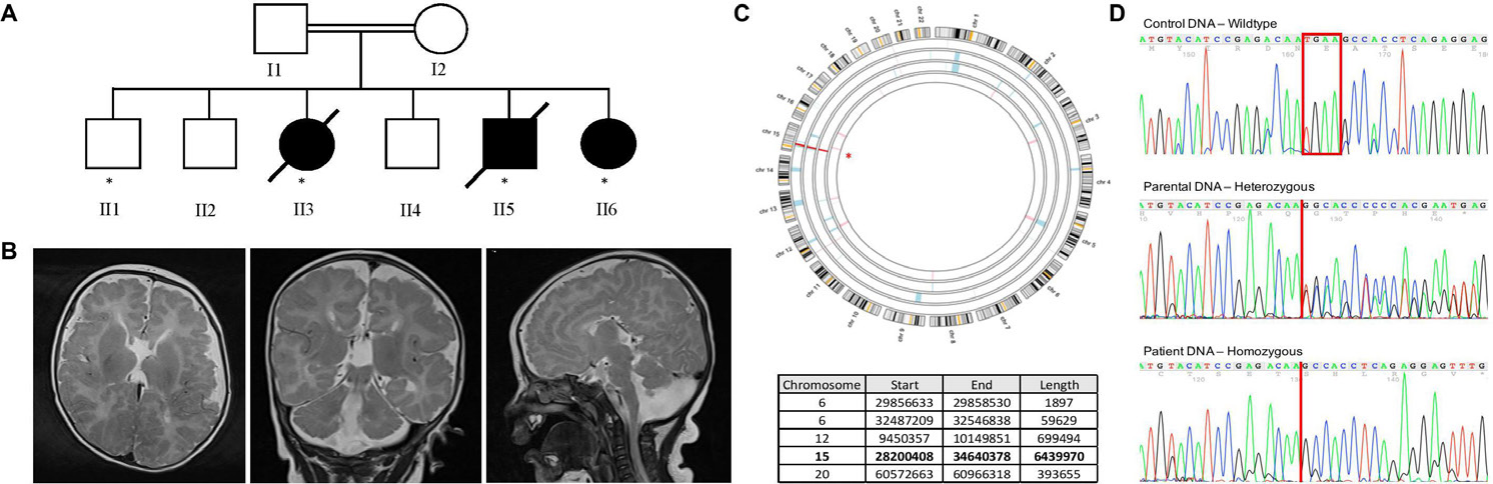
Identification and segregation of *HERC2* mutation. (A) Pedigree of the Libyan family outlining two generations. DNA samples from individuals marked with an asterisk (*) were processes using WES. (B) MRI scans from individual II:5 at 3 months of age. Axial T2-weighted image (left panel) showing reduced cerebral volume. The cortex is thickened bifrontally with loss of the grey/white matter interface and numerous interdigitations indicative of polymicrogyria. myelination is delayed with no evidence of myelination in the anterior limb of the internal capsule on either side, which is expected to be visible at this age. Corpus callosum agenesis is apparent and Sylvian fissures are slightly prominent. Coronal T2-weighted scan (middle) indicates cerebellar hypoplasia. Multiple small cerebellar folia and cerebellar polymicrogyria are apparent. Midline sagittal T2-weighted scan (right) shows considerable evidence of cerebellar, brainstem, pontine and medullary hypoplasias. Cerebral polymicrogyria and agenesis of the corpus callosum are also apparent. (C) Homozygosity mapping performed on the sample processes by WES, using the Agile Multi-Ideogram tool. Regions highlighted on the ideogram represent homozygous genomic intervals shared by the affected individuals only. Specific coordinates are presented on the table. (D) Variant confirmation using Sanger sequencing. Top electropherogram represents wild-type control DNA, and the box marks the four nucleotides that are deleted in the patients. The middle electropherogram is from parental DNA, with the line highlighting the breakpoint of the deletion. Heterozygous state of parental DNA shown here by the mismatch trace after the deletion point. The bottom panel is from affected individuals outlining the homozygous deletion and frameshift after the deletion point. WES, whole-exome sequencing.

WES was performed on genomic DNA from the three affected siblings alongside one unaffected sibling (marked with ‘*’ in figure 1A) from the family. Genomic DNA was diluted to 25 ng/µL and processed using the Agilent SureSelect QXT Target Enrichment kit according to the manufacturer’s protocol (Agilent Technologies, California, USA). DNA libraries were pooled and sequenced on an Illumina HiSeq 3000 using a 150 bp paired-end protocol.

### Bioinformatics analysis

The Burrows-Wheeler Aligner was used to align.fastq files to the human reference genome (GRCh37). The resulting bam file was further processed using Picard tools and the Genome Analysis Toolkit (GATK), according to best practice guidelines, to mark possible PCR duplicates and to check for accurate alignment around indels. Variants were called using HaplotypeCaller (GATK) and filtered against the National Center for Biotechnology Information’s dbSNP146, excluding any variants with a minor allele frequency (MAF) ≥1%. Variants were also excluded if present with a MAF of ≥1% in the Exome Aggregation Consortium (V.0.3.1) and Genome Aggregation Consortium (gnomAD, r2.0.2) databases. All remaining variants that segregated in an autosomal recessive mode of inheritance were annotated using Ensembl’s Variant Effect Predictor to predict functional consequences. Variants with a Combined Annotation Dependent Depletion (CADD) score of ≤15 were also excluded. Homozygosity mapping was performed using vcf files prior to filtering on dbSNP to identify homozygous regions shared by the affected individuals of the family using AgileMultiIdeogram (http://dna.leeds.ac.uk/agile/AgileMultiIdeogram/).

### PCR and Sanger sequencing

Segregation of the *HERC2* variant in the family was determined by PCR and Sanger sequencing using primers flanking exon 90 (forward: 5′-AGATGCACTTGAGGCTGACC, reverse: 5′-TGGAGCCAAATCCATTACTTT). PCR was performed using HotShot Master Mix protocol (Clent Life Sciences), and Sanger sequencing was carried out using BigDye Terminator V.3.1 kit (Life Technologies, California, USA) according to the manufacturer’s protocol. Sequencing was resolved on an ABI3130xl Sequencer (Life Technologies), and the electropherograms were analysed using 4Peaks software.

### Cell culture

Primary lines of human dermal fibroblasts (HDFs) were derived from skin biopsies of healthy and affected individual II:6 (figure 1A). Primary HDFs were cultured in Dulbecco’s Modified Eagle Medium: Nutrient Mixture F-12 (DMEM/F12) supplemented with 10% foetal calf serum at 37 °C/5%CO_2_.

### Western blotting

Whole-cell protein extracts were obtained from primary HDFs. Total soluble protein was collected using NP40-lysis buffer (50 mM Tris–HCl pH 8.0, 150 mM NaCl, 1% (v/v) NP-40, 1× protease/phosphatase inhibitors), and proteins were then separated on 7% NuPAGE Tris–acetate gels for higher molecular weight proteins. Proteins were transferred on to a methanol-activated polyvinylidene difluoride (PVDF) membrane for 5 hours at 40 mA at 4°C. Immunoblot analysis was performed using 1:1000 dilution of rabbit anti-HERC2 antibody (Bethyl Laboratories). Lower molecular weight proteins were separated on 4%–12% NuPAGE Bis–Tris sodium dodecyl sulfate–polyacrylamide gel electrophoresis gels and transferred onto PVDF membranes using 90 min transfer at 30mA. Immunoblot analysis was performed using 1:1000 dilution of rabbit anti-pericentriolar material 1 (PCM1) antibody (Proteintech Group), 1:500 of rabbit anti-Xeroderma pigmentosum complementation group A protein (XPA) antibody (a kind gift from Professor Majlinda Lako) and 1:1000 of rabbit anti-centrosomal protein 170kDa (CEP170) antibody (a kind gift from Professor Marius Ueffing). Primary antibodies were detected using a species-appropriate horseradish peroxidase (HRP)-conjugated antibody (Dako) at a 1:5000 dilution. Detection of HRP was achieved using the SuperSignal West Femto immunoblot detection system (Thermo Fisher Scientific).

### Immunofluorescence and confocal microscopy

Cells were seeded in 24-well plates on glass coverslips and 24 hours later fixed in ice-cold methanol for 5 min at −20°C. Fixed coverslips were blocked in 1% (w/v) non-fat milk solution and stained with mouse anti-MTCO2 (Abcam) at a final dilution of 1:1000. Appropriate secondary antibodies (Alexa Fluor, Thermo Fisher Scientific) and 4',6-diamidino-2-phenylindole (DAPI) were used at final dilutions of 1:1000. Confocal images were acquired with a Nikon A1R Confocal Microscope using NIS Elements software. Images were processed using FIJI software.

### Seahorse metabolism assays

Seahorse MitoStress and Glycolysis Stress tests were performed in three biological replicates on 96-well plates according to manufacturer’s instructions (Agilent Technologies). Primary fibroblasts obtained from a single affected child (II:6) and an age-matched healthy control (both passage number=3) were seeded in the Seahorse XF Cell Culture Microplate at a seeding density of 15 000 cells per well. For the MitoStress assay, the following toxins were used: 1.0 µM oligomycin, 2.0 µM carbonyl cyanide-4-(trifluoromethoxy)phenylhydrazone (FCCP) and 0.5 µM rotenone/antimycin A. For the glycolysis stress assay, the following toxins and substrates were used: 10.0 mM glucose, 1.0 µM oligomycin and 50.0 mM 2-deoxyglucose. At the end of each assay, the differences between the cell numbers in each well were normalised by histological staining with 1.0% (w/v) Crystal Violet, solubilisation with methanol and standard colorimetric quantification. We used Agilent Seahorse Wave Desktop software for data analysis.

### Statistical analysis

Normal distribution of data (Seahorse MitoStress and Glycolysis Stress tests, quantification of western blots) was confirmed using the Kolmogorov-Smirnov test (GraphPad Software). Pairwise comparisons were analysed by Student's two-tailed t-test using Prism V.7 (GraphPad Software). Results reported are from at least three independent biological replicates. Error bars on graphs represent SE of the mean (SEM).

## Results

### Clinical ascertainment

Three Libyan siblings with profound developmental delay were recruited to the study (figure 1A). Their parents were distantly related (third cousins). There was no known family history of similar developmental delay. None of the children had developed verbal or non-verbal communication skills, speech comprehension, head control or purposeful hand movements. None achieved ambulation. The children had structurally normal eyes but could not fix or follow; all were thought to be blind. All three children had choreiform movements and orofacial dyskinesias and were hypotonic and hypermobile. The affected boy had a horseshoe kidney and bilateral gross hydronephrosis with reduced renal cortex. None of the children had any specific facial dysmorphism, limb or cutaneous abnormalities. A urine metabolic screen for affected child II:3 did not reveal any specific findings, showing normal urine organic acid, acyl carnitine and amino acid levels. Serum lactate levels were normal (1.6 mmol/L) with no evidence of metabolic acidosis. All were gastrostomy fed. The younger two both suffered recurrent urinary tract infections. The two female children suffered recurrent seizures. II:3 died at the age of 7 years following a seizure, and II:4 died at 4 years due to respiratory failure.

All the affected siblings had MRI brain imaging. All had hypoplasia of the brainstem, and corpus callosum. All had signs of cortical migration abnormalities: II:3 with bilateral grey matter heterotopia, II5 with bifrontal polymicrogyria and potential cerebellar polymicrogyria, and II:6 with generalised polymicrogyria, including in the cerebellum (figure 1B). A summary of the clinical features observed in these affected individuals, as well as clinical features of all individuals reported previously with *HERC2* pathogenic mutations, are listed in table 1.

**Table 1:** Summary of clinical features for affected individuals with *HERC2* mutations

Publication and pedigree	Individual	Gender	Age seen (years)	Speech	Walked (years)	Intellectual disability	Childhood hypotonia	Seizures	Neuroimaging	Blindness	Genotype (NM_004667.5)
This report	II:3	F	6 (died 7)	N	N	++++	Y	Y	CMD, ACC	Y	c.13767_13770delTGAA p.(Asn4589LysfsTer4598)
	II:5	M	1.5 (died 4)	N	N	++++	Y	N	CMD, ACC	Y	
	II:6	F	3.5	N	N	++++	Y	Y	CMD, ACC	Y	
Harlalka *et al*: 1A^9^	VIII:7	F	39	100 words	4.3	++	Y	N	Nor	N	c.1781C>T, p.(Pro594Leu);
	VIII:8	M	35	<30 words	4.3	++/+++	Y	N	NK	N	
	IX:1	F	24	SS	4	++	Y	N	NK	N	
	IX:6	M	17	SS	4	++	Y	Y	NK	N	
	IX:7	F	19	SS	4	++	Y	N	Nor	N	
	X:1	F	13.6	<30 words	4	++	Y	N	Nor	N	
	X:2	F	12.6	10 words	4	++	Y	N	NK	N	
	X:4	F	5.1	N	N	++/+++	Y	N	ACC	N	
	X:5	F	6.7	SS	5	++	Y	N	NK	N	
	XI:2	M	7.8	Limited	3.5	+	Y	N	NK	N	
	XI:3	M	5.1	20 words	N	++	Y	Y	ACC	N	
Harlalka *et al*: 1B^9^	IX:8	M	17.8	SS	4.5	+/++	NK	Y	ACC	N	c.1781C>T, p.(Pro594Leu)
	IX:10	M	16.8	SS	4	+/++	NK	N	NK	N	
	IX:1	F	0.97	N	N	++	Y	Y	NK	N	
	IX:2	F	2.7	<10 words	N	+/++	Y	N	NK	N	
Puffenberger *et al* 8	Michigan sibship P1	NK	32	NK^a^	NK^b^	+/++/+++^c^	Y	N	NK	N	c.1781C>T, p.(Pro594Leu)
	Michigan sibship P2	NK	25	NK^a^	NK^b^	+/++/+++^c^	Y	N	NK	N	
	Michigan sibship P3	NK	31	NK^a^	NK^b^	+/++/+++^c^	N	N	NK	N	
	Wisconsin sibship P1	F	7	NK^a^	NK^b^	+/++/+++^c^	N	Y	NK	N	c.1781C>T, p.(Pro594Leu)
	Wisconsin sibship P2	F	2	NK^a^	NK^b^	+/++/+++^c^	N	Y	NK	N	
Puffernberger *et al* 8/Abraham *et al* 7	Puffenberger Ohio sibship P1/Abraham #1	F	Puffenberger: 40 Abrahams: 47	NK^a^	NK^b^	+/++/+++^c^	N	N	NK^d^	N	c.1781C>T, p.(Pro594Leu)
	Puffernberger Ohio sibship P2/Abraham #2	M	Puffenberger: 37 Abrahams: 43	NK^a^	NK^b^	+/++/+++^c^	N	N	NK^d^	N	
Abraham *et al* 7	#3	M	23	Few words	Nor	++	N	N	NK	NK	c.4625G>A, p.Arg1542His
	#4	F	11	10 words at 2 years, SS at 3 years	Nor	+	N	Y	N	N	
Morice-Picard *et al* 10	Proband	M	0, died 2 years	NK	NK	NK	Y	NK	ACC, CMD, megacisterna magna	NK (moderate retinal hypopigmentation)	chr15: g. 28143765_28429460 del including *OCA2* and *HERC2*
			Proportion with feature (where known)	Delayed speech: 20/20	Delayed walking: 18/20	Intellectual disability: 27/27	Childhood hypotonia: 19/26	Seizures: 9/27	Abn neuroimaging: 7/11	Blindness: 3/26	

NK^a^ means details of individual cases not known; most had words by 2 years.

NK^b^ means details of individual cases not known, walked at an average of 3.5 years (range 2.25–5.0 years).

+^c^ means details of individual cases not known, range mild–moderate/severe.

NK^d^ means details of individual cases not known, one sibling with normal MRI and one with mild cerebral atrophy.

+ indicates mild; ++ indicates moderate; +++ indicates severe; ++++ indicates profound.

Abn, abnormal; ACC, absent corpus callosum; CMD, cortical migration defect; F, female; M, male; N, no; NK, not known; Nor, normal; SS, short sentence; Y, yes.

### Whole-exome sequencing

Genomic DNA samples from the three affected individuals and an unaffected sibling were taken forward for WES library preparation using the Illumina QXT protocol, and sequencing was performed on an Illumina HiSeq 3000. Prior to data analysis,.fastq files and subsequent.bam files were assessed for sequence quality. An in-house bioinformatics pipeline was used for data analysis, assuming a recessive mode of inheritance, to filter out variants that were not compatible with Mendelian segregation based on the family’s pedigree.

To assist with variant filtration, autozygosity mapping was performed using the.vcf files of each sibling prior to SNP filtering. This revealed five regions of autozygosity shared between the three affected siblings but not the unaffected sibling. Using the described criteria, only a single homozygous frameshift variant c.13767_13770delTGAA in *HERC2* remained, which was present within the largest shared homozygous region (figure 1C). This frameshift is predicted to affect the protein from residue Asn4589, resulting in a premature termination codon p.(Asn4589LysfsTer4598). The variant has a CADD (V.1.3) score of 34 and is absent from dbSNP151 and gnomAD (V.2.1.1). Sanger sequencing confirmed the presence of the variant and appropriate segregation in all available family members (figure 1D).

### Effect of the HERC2 p.Asn4589LysfsTer4598 null allele on mitochondrial functions

**Figure 2:**
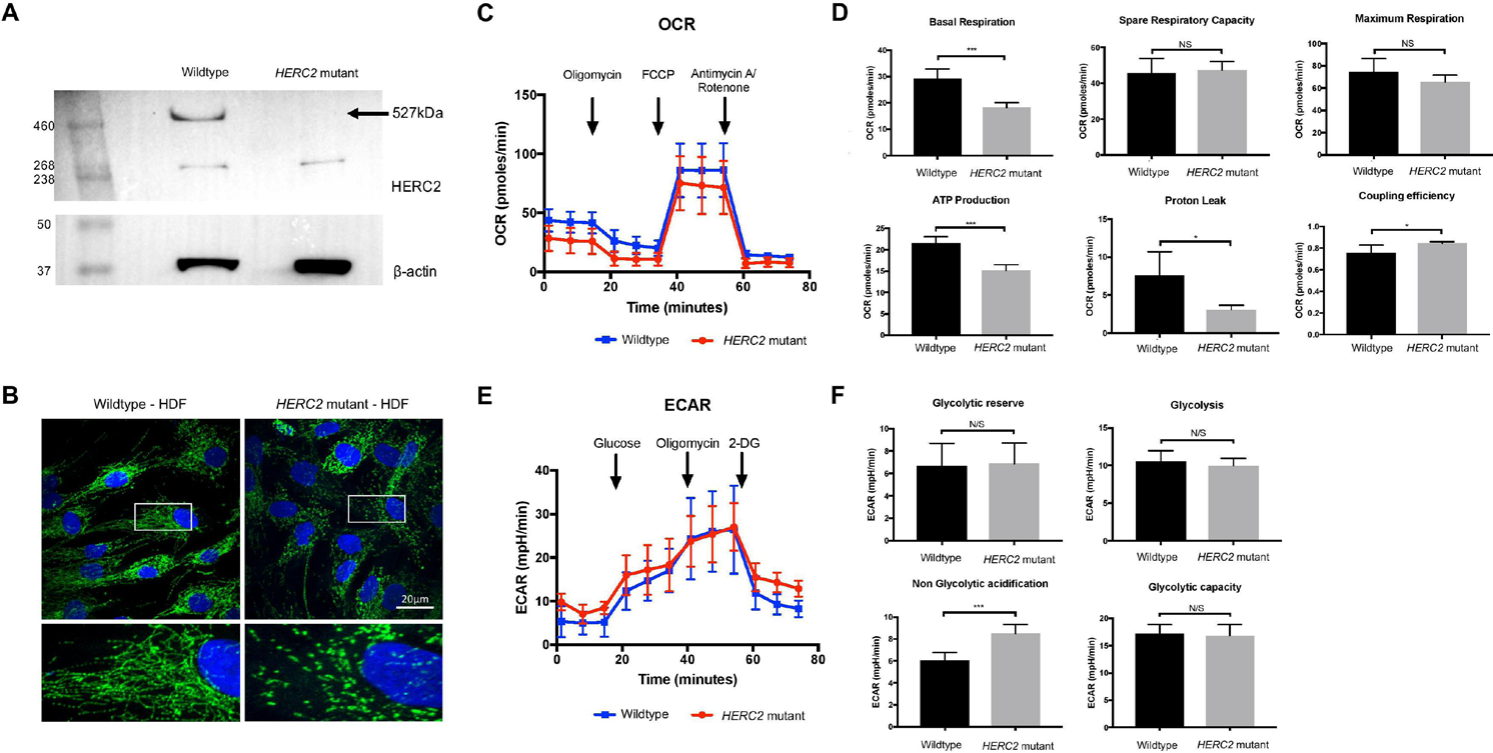
Loss of HERC2 protein and impact on mitochondrial function. (A) Western blot confirming loss of HERC2 protein. Protein extracted from wild type and *HERC2* mutant fibroblasts from affected individual II:6. (B) Immunofluorescence using 4',6-diamidino-2-phenylindole (DAPI) and MTCO2 antibody for mitochondrial imaging of wild type and *HERC2* mutant fibroblasts (magnification ×100). (C) Mitostress test using Seahorse XFe96 Analyzer. Real-time readings of OCR (pmol/min) are illustrated. (D) Key aspects of mitochondrial function are calculated using OCR values. Statistical tests were performed by a two-tailed Student t-test (n=3; NS; *p<0.05, ***p<0.001), and error bars indicate SEM. (E) Glycolysis stress test using Seahorse XFe96 Analyzer. Real-time readings of the ECAR (mpH/min) are illustrated. (F) Key aspects of glycolysis are calculated using ECAR values. Statistical tests were performed by a two-tailed Student t-test (n=3; NS; ***p<0.001), and error bars indicate SEM. 2-DG, 2-deoxyglucose; ECAR, extracellular acidification rate; NS, not significant; OCR, oxygen consumption rate.

To investigate the impact of the truncating mutation on the HERC2 protein, patient dermal fibroblasts were obtained from an affected individual (II:6) and soluble cellular protein extracted. Immunologically detectable HERC2 was absent in the insoluble cellular fraction after extraction (data not shown). A western blot against HERC2 revealed complete loss of full-length HERC2 protein as a result of the frameshift deletion (figure 2A) compared with control HDFs. Based on this result, the patient fibroblasts were proven to carry a homozygous *HERC2* null allele and were subsequently used to further characterise the impact of the mutation on cellular phenotypes and protein interactions.

The phenotype of severe delay, seizures and profound visual loss, muscle weakness and hypotonia observed in affected family members suggested that loss of HERC2 may affect mitochondrial function. Recent evidence also suggests that HERC2 mediates DNA repair and cell cycle regulation, critical to cellular homeostasis in the maintenance of mitochondrial dynamics and function.^11–13^ In addition, previous proteomic analysis on lymphoblastoid cell lines derived from patients with *HERC2* homozygous mutations p.Pro594Leu and p.Arg1542His, compared with control cell lines, revealed significant upregulation of mitochondrial dysfunction pathways, especially mitochondrial bioenergetics, suggesting a potential role for HERC2 in mitochondrial biology.^7^ Mitochondrial morphology was therefore assessed in control and patient HDFs by immunofluorescence microscopy. We observed striking mitochondrial fragmentation in the patient fibroblasts compared with the control (figure 2B).

To further elucidate the effect of HERC2 loss on mitochondrial function, the oxidative phosphorylation pathway and glycolytic pathway of energy production were tested using a Seahorse XFe96 Extracellular Flux Analyzer. Control and patient HDFs were challenged with inhibitors that affect different stages of each pathway and the oxygen consumption rate and extracellular acidification rate were measured before and after each inhibitor treatment, giving overall traces for the MitoStress and Glycolysis Stress assays, respectively (figure 2C, E). Measurements of oxidative phosphorylation function were determined from the traces in order to determine ATP production, basal and maximal respiration, proton leak and spare respiratory capacity (figure 2). Basal respiration, ATP production, proton leak and coupling efficiency were significantly disrupted in mutant *HERC2* cells compared with normal controls. Similarly, glycolytic pathway and glycolytic potential measurements showed that *HERC2* mutant fibroblasts from affected individual II:6 had a significantly increased non-glycolytic acidification level compared with the normal control fibroblasts (figure 2F). This suggested a source of acidification separate to the glycolysis pathway since the levels of glycolysis, glycolytic capacity and glycolytic reserve were indistinguishable between the two cell-lines.

### Effect of the HERC2 p.Asn4589LysfsTer4598 null allele on protein–protein interactions

**Figure 3:**
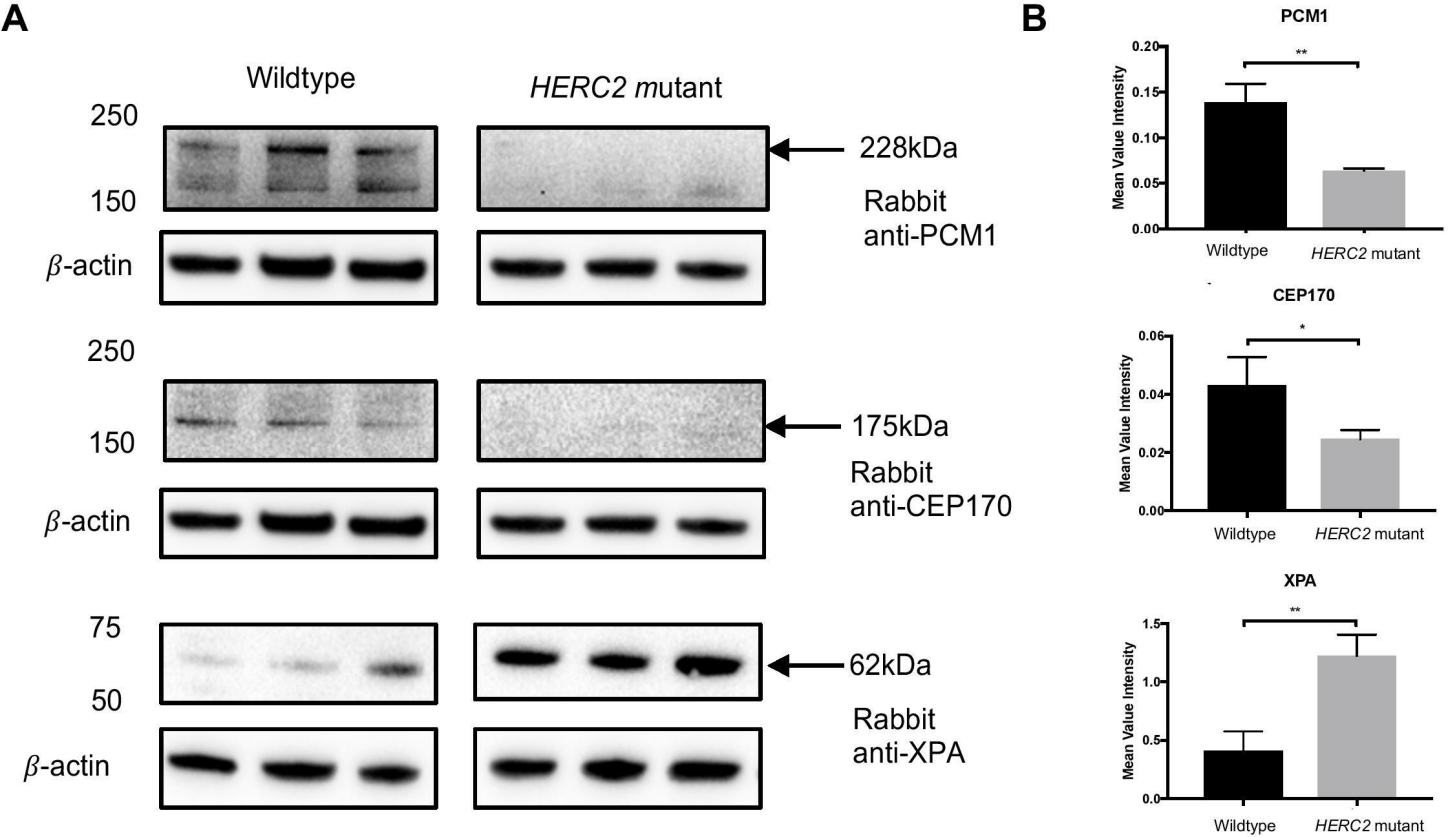
Loss of HERC2 affects other interacting partners. (A) Representation of protein levels on western blot membranes for the interacting proteins PCM1, CEP170 and XPA compared with loading control β-actin. Western blots include all three biological replicates of each experiment. (B) Bar graphs quantitating the results from all three biological replicates normalised to β-actin. All statistical tests were performed by a two-tailed Student t-test (n=3; NS; *p<0.05, **p<0.01). Error bars indicate SEM. PCM1, pericentriolar material 1.

The impact of complete loss of HERC2 on potential interacting and target proteins was also investigated. Interacting proteins were chosen from either UniProt interaction data or manual collation of the primary literature.^4 6^ Since HERC2 is an E3 ubiquitin-ligase protein, we hypothesised that HERC2 loss would cause an increase in protein levels of XPA as it is considered to be a potential substrate tagged for degradation. HERC2 has also been reported to be a modulator of centrosome architecture^11^ and is implicated in cell cycle regulation and mitosis. Both CEP170 and PCM1 are centrosomal proteins that are annotated as both hits and baits in reciprocal affinity capture-mass spectrometry (MS) interaction studies of HERC2 (see BioGRID3.5 result summary, https://thebiogrid.org/114438) and that both affect downstream cell cycle-dependent processes.^6 14 15^ CEP170 and PCM1 also interact with the same RCC1 domain as UBE3A, potentially sharing a cellular mechanism that, when disrupted, causes Angelman-like or more severe developmental phenotypes.^16^ Soluble protein was extracted from control and patient HDFs and the protein levels of XPA, CEP170 and PCM1 were quantified by western blot. This revealed significant changes in the protein levels of all three interacting proteins (figure 3). As predicted, intracellular XPA levels were significantly increased in affected HDFs compared with normal healthy control HDFs. Conversely, intracellular levels of CEP170 and PCM1 were significantly decreased in affected HDFs compared with the controls.

## Discussion

Loss of HERC2 is likely to affect many cellular processes, specifically cell cycle regulation and DNA repair mechanisms, resulting in cellular stress and defective cellular homeostasis as a downstream consequence.^11 12^ Unbalanced homeostasis can be critical in mitochondrial dynamics and function. This was confirmed by the observation of striking fragmentation of the mitochondrial network observed in mutant fibroblasts compared with normal controls, which is a key feature of mitochondrial stress. This observation was further examined by live cell metabolic assays, evaluating both the oxidative phosphorylation and the glycolysis pathways. Measurements from real-time traces revealed significantly reduced basal respiration levels and ATP production in the *HERC2*-mutated cells from affected individual II:6, indicative that the mutant cells have switched their energy production to other less efficient means than oxidative phosphorylation.^17^ One of the reasons for doing so could be a defect in ionic homeostasis due to altered proton leak in the mitochondria, as observed following treatment with the decoupler FCCP, leading to reduced proton-motive force and aerobic ATP production.

For the glycolysis stress assay, the level of glycose-to-pyruvate conversion was very similar between normal control and affected HDFs. In addition, there was no significant difference between the glycolytic capacity and glycolytic reserve, indicating that under normal conditions, the levels of glycolysis in both cell types were the same and, when forced, both can reach maximum glycolysis. A significant increase was observed, however, in non-glycolytic acidification levels in mutant fibroblasts from the affected individual II:6 compared with controls. This observation implies a source of acidification other than the glycolysis pathway, potentially arising from the conversion of pyruvate to lactate in order to maintain energy requirements for *HERC2*-mutated cells.^18^ The observation of defective mitochondrial bioenergetics, specifically the oxidative phosphorylation pathway, supports the findings of Abraham *et al* that suggest HERC2 has a key role in mitochondrial function.^7^

Due to the multiple functions of HERC2, principally as an E3 ubiquitin protein ligase, the complete loss of the protein is expected to have an impact on interactants and substrates. To test this hypothesis, we examined the protein levels of various known substrates in HDFs from affected individual II:6. Levels of XPA, a known HERC2 interactant,^2^ were increased in *HERC2*-mutated cells compared with the controls, supporting the hypothesis that XPA is a substrate tagged for degradation by HERC2 and that is consistent with the findings in a previous study.^10^ Loss of HERC2 therefore results in incorrect turnover of XPA,^2^ which could critically affect downstream DNA repair mechanisms or promote a pathogenic outcome due to aggregation of protein complexes.

Roles for HERC2 have also been described in mitosis, spindle formation and cell cycle regulation by interacting with centrosomal and cell cycle regulating proteins or ubiquitinating these proteins for specific signalling purposes,^6 19 20^ including PCM1 and CEP170. PCM1 is a component of centriolar satellites and is essential for centrosome assembly and function by correctly anchoring microtubules to the centrosome.^21^ PCM1 is also involved in regulating primary cilia disassembly before entering mitosis.^12^ CEP170 (centrosomal protein 170 kDa) is another component of the centrosome but is specifically localised to the mother centriole at the subdistal appendages, as well as in spindle formation and maintenance of microtubular organisation.^22^ Both of these proteins are key regulators of cell cycle-dependent events and are both significantly reduced in patient fibroblasts, suggesting a potential systemic defect in cell cycle regulation and cell division. These observations provide a potential explanation for the severe neurodevelopmental phenotypes observed in these patients.

To date, only two pathogenic *HERC2* missense variants have previously been reported (p.Pro594Leu and p.Arg1542His). Twenty-two individuals homozygous for p.Pro594Leu have been reported with what is described as an autism spectrum disorder that has phenotypic overlap with Angelman syndrome.^8^ All reported cases had speech and language delay where data were available (15/15), walking delay (22/22) and intellectual disability (22/22) varying from mild to severe, but with the majority being mild/moderate.^7–9^ Childhood hypotonia was reported in 15/20 with the p.Pro594Leu missense mutation. Two individuals homozygous for p.Arg1542His have also been reported, affected with similar delays in speech and language development and intellectual disability to affected individuals with p.Pro594Leu, but without the walking delay or childhood hypotonia.^7^ Seizures are not a consistent feature, reported in 6/22 of the p.Pro594Leu cases and 1/2 of the p.Arg1542His cases. What is clear is that the cases we describe in the present report have a much more profound developmental phenotype than those previously reported with missense mutations. All the siblings in the present report have profound intellectual disability, no verbal or non-verbal communication skills, and no head control or purposeful movements. In addition, all affected individuals were blind, had involuntary choreiform movements and signs of cortical migration defects on neuroimaging not previously observed in other *HERC2*-mutated cases. Two out of three had seizures and the two elder of the three siblings had passed away at the time of ascertainment, aged 4 and 7 years. None of the previously reported affected individuals carrying missense mutations have died, with most being much older than the siblings described in the present report.

A homozygous 286 kb deletion that spans *HERC2* and *OCA2* has also been reported, in which a much more severe form of developmental delay and lethality was described.^10^ This is the only other report of an ocular phenotype in a *HERC2*-mutated individual, comprising moderate retinal hypopigmentation. However, this is consistent with deletion of the *OCA2* gene, mutations which are known to cause non-syndromic oculocutaneous albinism type 2 (MIM 203200). The deletion resulted in a HERC2-OCA2 fusion protein and complete loss of both HERC2 and OCA2. The complete loss of a large and important E3 ligase will have a detrimental effect on diverse cellular processes, including DNA damage repair and cell cycle regulation, which is also supported by the observation that the *Herc2* homozygous null mouse is embryonically lethal.^20^

In summary, we have identified a family with a severe neurodevelopmental disorder due to complete loss of HERC2 and show that this has a significant impact on mitochondrial biogenesis and function, in addition to effects on the levels of known substrate proteins. This work establishes, for the first time, a clear genotype–phenotype correlation for the complete loss of HERC2. We suggest that HERC2 should be included in gene panels for non-specific severe neurodevelopmental disorders, in order to improve the sensitivity of diagnostic testing for patients with developmental delay but without a molecular diagnosis. This is important for the future stratification and management of inherited disorders with non-syndromic, profound developmental delay because, without a molecular diagnosis, these conditions are often clinically indistinguishable.

## Data Availability

All data relevant to the study are included in the article or uploaded as supplementary information. Further details and data can be obtained from the corresponding author CAJ (email: c.johnson@leeds.ac.uk).
